# Thorax support vest to prevent sternal wound infections in cardiac surgery patients—a systematic review and meta-analysis

**DOI:** 10.1093/icvts/ivae055

**Published:** 2024-03-26

**Authors:** Tulio Caldonazo, Michele Dell’Aquila, Gianmarco Cancelli, Lamia Harik, Giovanni Jr Soletti, Johannes Fischer, Hristo Kirov, Mohamed Rahouma, Mudathir Ibrahim, Michelle Demetres, Kevin R An, Leonard Girardi, Torsten Doenst, Mario Gaudino

**Affiliations:** Department of Cardiothoracic Surgery, Jena University Hospital, Friedrich-Schiller-University, Jena, Germany; Department of Cardiothoracic Surgery, Weill Cornell Medicine, New York, NY, USA; Department of Cardiothoracic Surgery, Weill Cornell Medicine, New York, NY, USA; Department of Cardiothoracic Surgery, Weill Cornell Medicine, New York, NY, USA; Department of Cardiothoracic Surgery, Weill Cornell Medicine, New York, NY, USA; Department of Cardiothoracic Surgery, Weill Cornell Medicine, New York, NY, USA; Department of Cardiothoracic Surgery, Jena University Hospital, Friedrich-Schiller-University, Jena, Germany; Department of Cardiothoracic Surgery, Jena University Hospital, Friedrich-Schiller-University, Jena, Germany; Department of Cardiothoracic Surgery, Weill Cornell Medicine, New York, NY, USA; Department of General Surgery, Maimonides Medical Center, Brooklyn, NY, USA; Nuffield Department of Surgical Sciences, University of Oxford, Oxford, UK; Samuel J. Wood Library & CV Starr Biomedical Information Center, Weill Cornell Medicine, New York, NY, USA; Department of Cardiothoracic Surgery, Weill Cornell Medicine, New York, NY, USA; Division of Cardiac Surgery, Department of Surgery, University of Toronto, Toronto, ON, Canada; Department of Cardiothoracic Surgery, Weill Cornell Medicine, New York, NY, USA; Department of Cardiothoracic Surgery, Jena University Hospital, Friedrich-Schiller-University, Jena, Germany; Department of Cardiothoracic Surgery, Weill Cornell Medicine, New York, NY, USA

**Keywords:** Sternal wound infection, Cardiac surgery, Thorax support vest, Stability, Osteomyelitis

## Abstract

**OBJECTIVES:**

Midline sternotomy is the main surgical access for cardiac surgeries. The most prominent complication of sternotomy is sternal wound infection (SWI). The use of a thorax support vest (TSV) that limits thorax movement and ensures sternal stability has been suggested to prevent postoperative SWI.

**METHODS:**

We performed a meta-analysis to evaluate differences in clinical outcomes with and without the use of TSV after cardiac surgery in randomized trials. The primary outcome was deep SWI (DSWI). Secondary outcomes were superficial SWI, sternal wound dehiscence, and hospital length of stay (LOS). A trial sequential analysis was performed. Fixed (*F*) and random effects (*R*) models were calculated.

**RESULTS:**

A total of 4 studies (3820 patients) were included. Patients who wore the TSV had lower incidence of DSWI [odds ratio (OR) = *F*: 0.24, 95% confidence interval (CI), 0.13–0.43, *P* < 0.01; *R*: 0.24, 0.04–1.59, *P* = 0.08], sternal wound dehiscence (OR = *F*: 0.08, 95% CI, 0.02–0.27, *P* < 0.01; *R*: 0.10, 0.00–2.20, *P* = 0.08) and shorter hospital LOS (standardized mean difference = *F*: −0.30, −0.37 to −0.24, *P* < 0.01; *R*: −0.63, −1.29 to 0.02, *P* = 0.15). There was no difference regarding the incidence of superficial SWI (OR = *F*: 0.71, 95% CI, 0.34–1.47, *P* = 0.35; *R*: 0.64, 0.10, 4.26, *P* = 0.42). The trial sequential analysis, however, showed that the observed decrease in DSWI in the TSV arm cannot be considered conclusive based on the existing evidence.

**CONCLUSIONS:**

This meta-analysis suggests that the use of a TSV after cardiac surgery could potentially be associated with a reduction in sternal wound complications. However, despite the significant treatment effect in the available studies, the evidence is not solid enough to provide strong practice recommendations.

**Figure:**
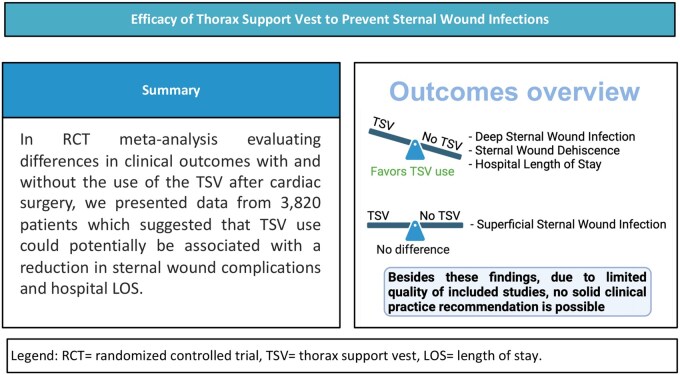

## INTRODUCTION

Currently, midline sternotomy is a widespread method for direct thorax access [1], and despite advancements in minimally invasive surgery, the majority of complex cardiac procedures are performed through midline sternotomy. Albeit rare (0.5–5%), complications of midline sternotomy such as sternal wound infection (SWI), sternal wound dehiscence, repeat sternotomy, mortality, prolonged hospital stay and increased hospital costs were described in different patient series [2–4].

In order to prevent post-sternotomy complications, several intra- and extra-thoracic strategies have been proposed [5–7]. In recent years, devices such as chest blinders and thorax support vests (TSV) have been applied to optimize post-sternotomy recovery. The TSV provides sternal support after cardiac surgery by stabilizing the surgical site, minimizing pressure on the suture lines and limiting thoracic movement. The TSV aims to improve surgical wound healing and to reduce SWI, sternal wound dehiscence and pain during respiration and in the rehabilitation process.

This meta-analysis and systematic review was performed to compare clinical outcomes with and without the use of TSV following midline sternotomy.

## METHODS

Institutional Review Board approval of this analysis was not required as no human or animal subjects were involved. The present review was registered with the National Institute for Health Research International Registry of Systematic Reviews (PROSPERO, CRD42023486461). This article follows the Preferred Reporting Items for Systematic Reviews and Meta-Analyses (PRISMA) guidelines.

### Search strategy

A medical librarian (Michelle Demetres) performed a comprehensive literature search to identify randomized clinical trials (RCTs) reporting clinical outcomes in patients who underwent cardiac surgery via midline sternotomy and used a TSV postoperatively, and those who do not use it. Searches were performed in August 2023 and included three databases: Ovid MEDLINE (all; 1946 to present), Ovid EMBASE (1974 to present) and Cochrane Library. The complete search strategy for Ovid MEDLINE is available in Supplementary Material, Table S1.

### Study selection and data extraction

After de-duplication, studies were screened by two independent reviewers (Tulio Caldonazo and Michele Dell’Aquila) and discrepancies were resolved by the senior author (Mario Gaudino). A first round of screening based on title and abstract content was performed and studies were considered for inclusion if they were written in English and compared outcomes between patients who underwent cardiac surgery via midline sternotomy and used a TSV postoperatively and those who did not use a TSV. Animal studies, abstracts, case reports, commentaries, editorials, expert opinions, conference presentations, studies with paediatric populations, and studies not reporting the outcomes of interest were excluded. The individual studies’ eligibility criteria are displayed in Supplementary Material, Table S2. The full text was pulled for the selected studies for a second round of eligibility screening. References for articles selected were also reviewed for relevant studies not captured by the original search.

Two investigators (Tulio Caldonazo and Michele Dell’Aquila) independently performed data extraction and the integrity was verified by the corresponding author (Mario Gaudino). The extracted variables included study characteristics (publication year, country, sample size, study design, mean follow-up and population adjustments) as well as patient demographics [age, sex, body mass index, diabetes, smoking status, chronic renal failure, chronic obstructive pulmonary disease (COPD), peripheral vascular disease (PVD) and prior myocardial infarction]. The quality of the included studies was assessed using the Cochrane risk-of-bias tool for RCTs (Supplementary Material, Table S3).

### Outcomes

The primary outcome was deep sternal wound infection (DSWI) [defined as Centers for Disease Control and Prevention (CDC) II and III wound infection per CDC guidelines [8]]. Secondary outcomes were superficial sternal wound infection (SSWI) (defined as CDC I wound infection), sternal wound dehiscence and hospital LOS. Data regarding perioperative mortality was scarce. These perioperative outcomes were defined by in-hospital/30-day outcomes.

### Statistical analysis

Categorical values were analysed using odds ratio (OR) and 95% confidence intervals (CIs). An OR >1 indicated that the outcome was more frequently present in the TSV group. Continuous variables were analysed using standardized mean difference (SMD) and 95% CI. An SMD of more than zero corresponded to longer stay in the TSV group.

Random effects models were used. Results were displayed in Forest plots. Between-study statistical heterogeneity was assessed with the Cochran *Q* statistic and by estimating *I*^2^. High heterogeneity was defined as a significance level of *P* < 0.10 and *I*^2^ of at least 50% or more.

### Sensitive analyses

All the meta-analytic comparisons were repeated using fixed models as sensitivity analyses. A sub-group and leave-one-out analysis for the primary outcome was performed to assess the robustness of the estimate. A funnel plot was performed to address publication bias. Meta-regression was used to explore the association of age, chronic renal failure, COPD and PVD with the pooled estimate for the primary outcome.

A trial sequential analysis (TSA) was performed to weigh type I and II errors. Briefly, TSA is a newly introduced cumulative frequentist meta-analysis approach, designed to assess type I and II errors and determine when the observed effect size is sufficiently robust against potential influences from additional studies. Despite its foundation in frequentist principles, utilizing *P* values and methods for type I and type II errors, TSA also integrates aspects of Bayesian reasoning. Notably, the determined sample size in TSA is linked to the combined effect size estimated in a meta-analysis [9]. The crossover of the *Z* score and the statistical significance boundary corresponds to two-sided *P*-value of 0.05 meaning that the result of the comparison is significant. All statistical analyses were performed using R (version 4.1.1, R Project for Statistical Computing) within RStudio.

## RESULTS

### Study characteristics

**Figure 1:**
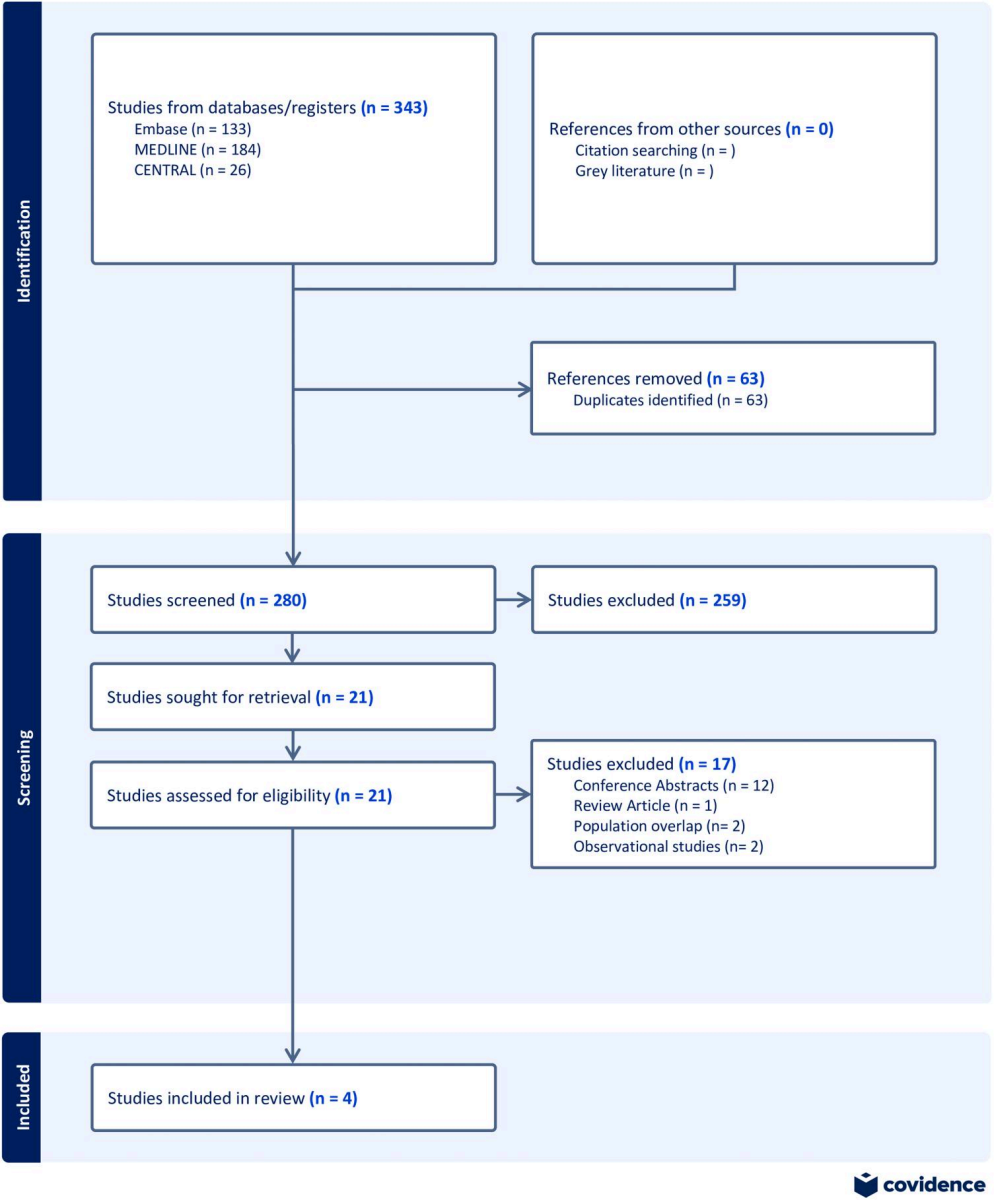
Preferred Reporting Items for Systematic Reviews and Meta-Analyses (PRISMA) flow diagram.

A total of 343 studies were screened, of which 4 met the criteria for inclusion in the final analysis. Figure 1 shows the PRISMA flowchart for study selection. Included studies were published between 2011 and 2016. The studies originated from Italy, Turkey, Austria and Germany.

A total of 3820 patients were included in the final analysis, 1986 of whom received the TSV. The number of patients in each study ranged from 221 to 2539, with a median sample size of 530 (IQR = 1379). Table 1 shows the details of the included studies.

**Table 1:** Summary of included studies.

Author	Year of publication	Country	Number of patients	Study design	Mean follow-up	Extracted outcomes
Caimmi *et al.* [20]	2016	Italy	310 TSV 155 No TSV 155	Randomized, single centre	2.4 years	Sternal wound dehiscence Hospital length of stay
Celik *et al.* [21]	2011	Turkey	221 TSV 100 No TSV 121	Randomized, single centre	6 months	Deep sternal wound infection Superficial sternal wound infection Sternal wound dehiscence Hospital length of stay
Gorlitzer *et al.* [22]	2013	Austria	2539 TSV 1351 No TSV 1188	Randomized, multicentre	90 days	Deep sternal wound infection Superficial sternal wound infection Hospital length of stay
Tewarie *et al.* [23]	2012	Germany	750 TSV 380 No TSV 370	Randomized, single centre	8 weeks	Deep sternal wound infection Superficial sternal wound infection Sternal wound dehiscence Hospital length of stay

TSV: thorax support vest.

### Patient characteristics

The mean age ranged from 63.9 to 70.4 years; the percentage of female patients ranged from 30.0% to 70.8%; the mean body mass index ranged from 27.9 to 30.0 kg/m^2^; the percentage of diabetes ranged from 29.5% to 48.5%; the percentage of chronic renal failure ranged from 8.0% to 14.9%; the percentage of COPD ranged from 19.7% to 63.0%; and the percentage of PVD ranged from 11.1% to 40.0%. The data regarding smoking status and prior myocardial infarction were sparse. Supplementary Material, Table S4 summarizes the demographic data of the patient population in each study.

### Meta-analysis—primary outcome

**Figure 2:**
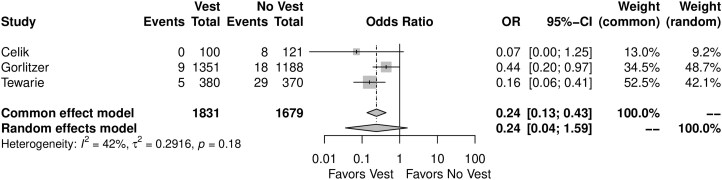
Forest plot for the primary end point (deep sternal wound infection). CI: confidence interval; OR: odds ratio.

When compared with the group who did not wear the TSV, the patients who used the TSV showed lower incidence of DSWI (OR = *F*: 0.24, 95% CI, 0.13–0.43, *P* < 0.01; *R*: 0.24, 0.04–1.59, *P* = 0.08). Figure 2 shows the Forest plot for DSWI. The leave-one-out analysis was consistent with the main analysis (Supplementary Material, Fig. S1). Supplementary Material, Fig. S2 shows the funnel plot.

On meta-regression, none of the addressed variables showed to be associated with higher ORs for the primary outcome (Supplementary Material, Table S5).

**Figure 3:**
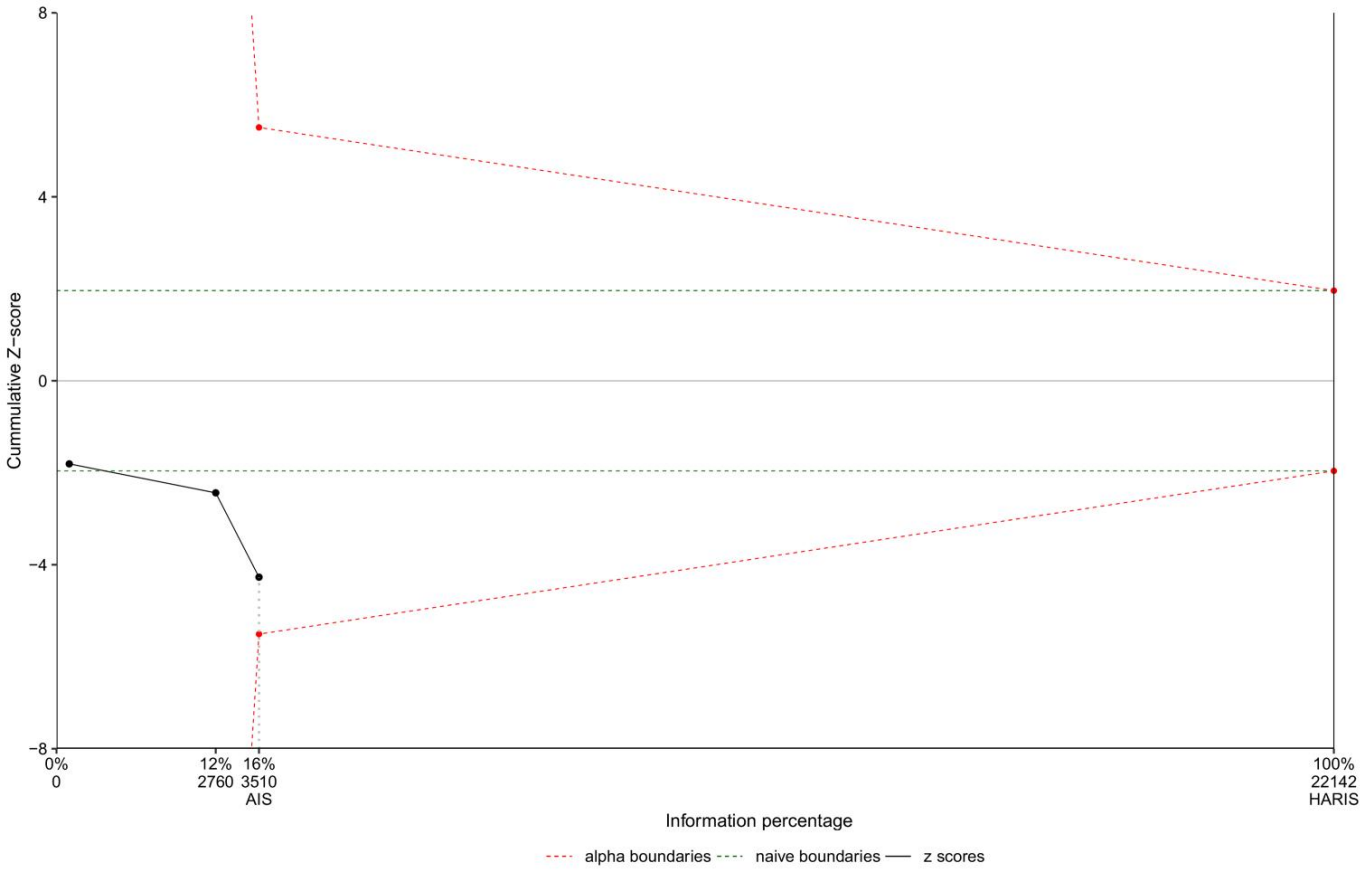
Trial sequential analysis for the primary endpoint (deep wound sternal infection). The *Z* score (solid line with three dots) crossed the conventional statistical significance boundary (dashed central line) that corresponds to two-sided *P*-value of 0.05 meaning that the result of the comparison is significant. While the number of included patients [achieved information size (AIS)] was 3510, heterogeneity adjusted required information size (HARIS) revealed a required sample size of 22 142. The *Z* score (periphery solid line) did not cross HARIS (vertical black line on right side).

The trial sequential showed that the observed decrease in DSWI in the TSV arm cannot be considered conclusive based on the existing evidence (Fig. 3).

### Secondary outcomes

**Figure 4:**
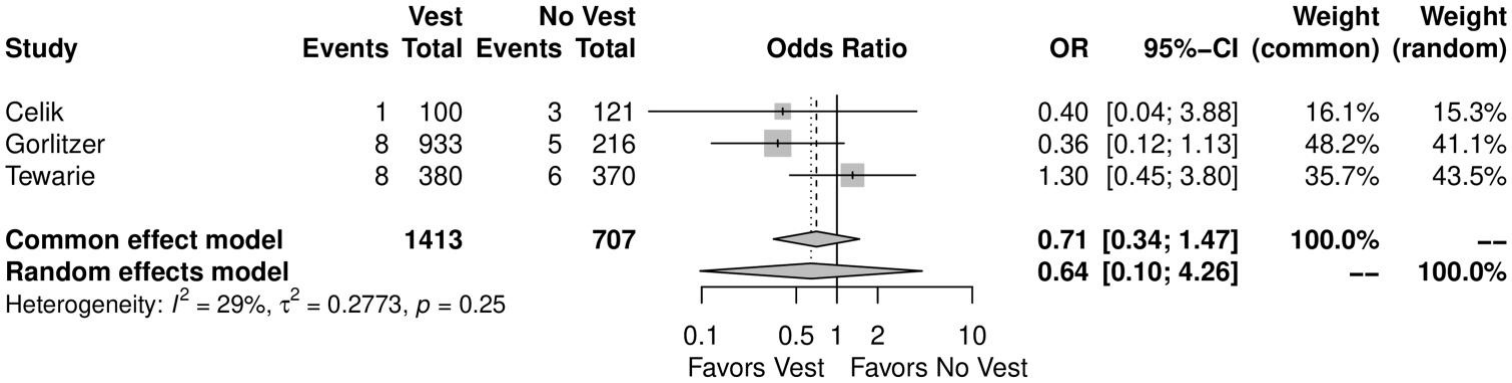
Forest plot for the superficial sternal wound infection. CI: confidence interval; OR: odds ratio.

Table 2 outlines the detailed results of the meta-analysis. There was no significant difference in the incidence of SSWI between the use of the TSV and no TSV (OR = *F*: 0.71, 95% CI, 0.34–1.47, *P* = 0.35; *R*: 0.64, 0.10, 4.26, *P* = 0.42, Fig. 4).

**Table 2:** Outcomes summary.

Outcome	Number of studies	Number of patients	Effect estimate (95% CI, *P*-value) Fixed (*F*) and random (*R*) effects model	Heterogeneity (*I*^2^, *P*-value)
Deep sternal wound infection	3	2120	*F*: OR = 0.24, 95% CI, 0.13–0.43, *P* < 0.01 *R*: OR = 0.24, 95% CI, 0.04–1.59, *P* = 0.08	*I* ^2^ = 42%, *P* = 0.18
Superficial sternal wound infection	3	2120	*F*: OR = 0.71, 95% CI, 0.34–1.47, *P* = 0.35 *R*: OR = 0.64, 95% CI, 0.10, 4.26, *P* = 0.42	*I* ^2^ = 29%, *P* = 0.25
Sternal wound dehiscence	3	1281	*F*: OR = 0.08, 95% CI, 0.02–0.27, *P* < 0.01 *R*: OR = 0.10, 95% CI, 0.00–2.20, *P* = 0.08	*I* ^2^ = 11%, *P* = 0.32
Hospital length of stay	4	3820	*F*: SMD = −0.30, 95% CI, −0.37 to −0.24, *P* < 0.01 *R*: SMD = −0.63, 95% CI, −1.29 to 0.02, *P* = 0.15	*I* ^2^ = 98%, *P* < 0.01

CI: confidence interval; OR: odds ratio; SMD: standard mean difference.

**Figure 5:**
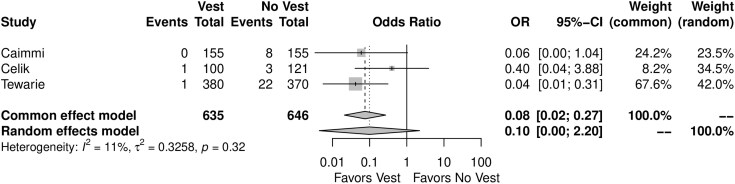
Forest plot for sternal wound dehiscence. CI: confidence interval; OR: odds ratio.

When compared with the group who did not wear the TSV, the patients who used the TSV showed lower incidence of sternal wound dehiscence (OR = *F*: 0.08, 95% CI, 0.02–0.27, *P* < 0.01; *R*: 0.10, 0.00–2.20, *P* = 0.08, Fig. 5).

**Figure 6:**
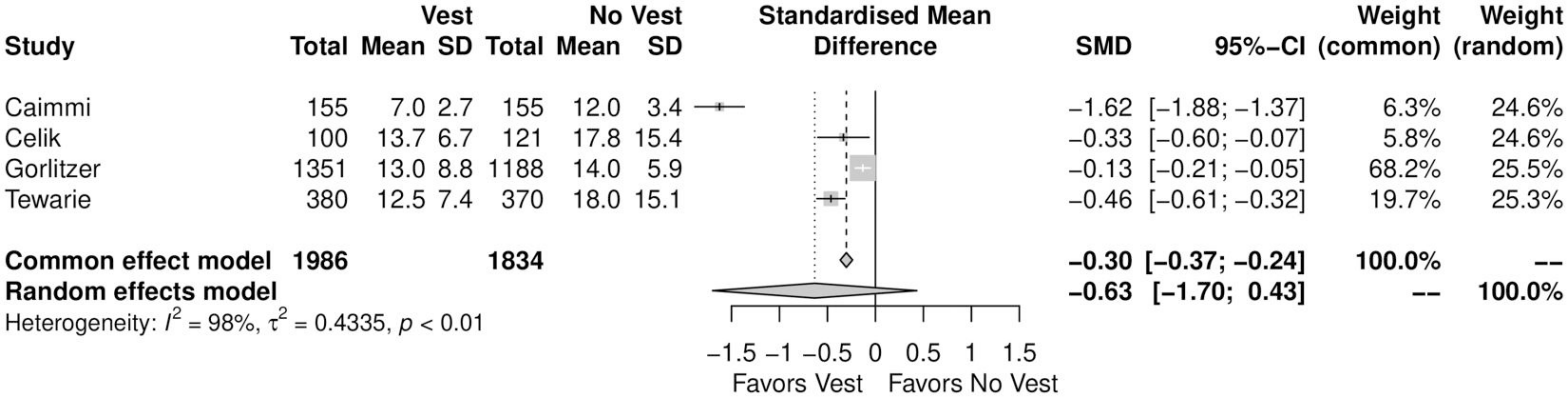
Forest plot for the hospital length of stay. CI: confidence interval; SMD: standardized mean difference.

When compared with the group who did not wear the TSV, the patients who used the TSV showed shorter hospital LOS (SMD = *F*: −0.30, −0.37 to −0.24, *P* < 0.01; *R*: −0.63, −1.29 to 0.02, *P* = 0.15) (Fig. 6).

## DISCUSSION

This meta-analysis of 3820 patients found that the use of a TSV after cardiac surgery via midline sternotomy was associated with lower incidence of DSWI, sternal wound dehiscence and shorter hospital LOS. No significant difference was found between the two groups regarding SSWI.

This is the first meta-analysis to address post-sternotomy complications, comparing clinical outcomes based on the use of TSV. Three of the four included articles reported the primary outcome of interest (DSWI) and all the studies included at least 1 secondary outcome. Overall, all the studies reporting DSWI and sternal wound dehiscence demonstrated individually significant lower rates for patients wearing a TSV, following sternotomy.

A previous descriptive systematic review concluded that early post-sternotomy use of an external non-elastic sternal support device would reduce overall sternal wound complications and may reduce the hospital LOS [10]. In this context, observational series showed also similar findings as the current analysis [11, 12].

SWIs have considerable clinical and economic implications, and thus, the American Association for Thoracic Surgery established guidelines for treatment and prevention of post-sternotomy complications only in 2016 [6, 13]. SSWI incidence rate is 1.1–6.7%, whereas the incidence of DSWI ranges from 0.1% to 3.7% [14, 15]. Although these incidence rates are slightly similar, the morbidity and mortality rates differ dramatically (i.e. 0.5–8% for SSWI and ∼47% for DSWI [14]).

Currently, there are several methods for the treatment of SWIs. The conventional procedures are surgical debridement, closed suction antibiotic catheter irrigation systems, negative pressure therapy and flap coverages [14]. In this context, it is possible to adopt preventive measures preoperatively, intraoperatively and postoperatively. Prioritizing patient health and education prior to sternotomy, encompassing risk factors such as diabetes, obesity, and smoking, would reduce post-sternotomy complications. From a clinical perspective, effective antibiotic prophylaxis, optimal surgery duration and sternal wound closure could contribute to complications reduction. In the postoperative phase, timely wound care, infection monitoring and follow-up could improve patients’ recovery trajectory [13].

Further preventive measures could be considered for specific surgical procedures. For instance, the majority of the patients in the articles analysed underwent coronary artery bypass graft (CABG). In CABG, conduit selection and harvesting represent modifiable variables which appear to have an impact on DSWI incidence. In the Arterial Revascularization Trial [16] and in other observational studies [17–19], the use of Bilateral Internal Thoracic Artery grafts as conduit for CABG was associated with an increased incidence of DSWI, which was associated with a higher risk of long-term mortality. For this reason, using a TSV could potentially improve clinical outcomes in patients at high risk for DSWI who undergo CABG with Bilateral Internal Thoracic Artery grafts.

Taken together, our analysis suggests (in a preliminary assessment) that the application of TSV can contribute to the reduction of sternal wound complications, specifically, DSWI and sternal wound dehiscence. However, the critical analysis of the pooled estimated effect of these trials has shown that more data are needed to really demonstrate a beneficial effect of the use of TSV on the primary end point. In other words, the current sample size of the studies included in the analysis is extremely small compared to the ideal sample size (11 946 patients) to conclude an effect of this magnitude on the primary end point.

Finally, besides the necessity of more data for the adequate power, remains important the fact that further clinical research and standard clinical protocols are necessary to better understand the efficacy of this device in order to ameliorate clinical outcomes post-sternotomy.

### Study strengths and limitations

This is the first meta-analysis to address this important topic. Moreover, we analysed 4 different outcomes and performed different sensitive analyses. However, this work has limitations such as the lack of mortality data and, above all, the lack of long-term end points. The fact that different studies present populations with different risk factors for SW infections results in a certain inter-study heterogeneity, which can influence the results. In addition, blinding of participants of the RCTs included in the analysis was not possible, and Tewarie *et al.* did not provide a clear description of the randomization process. Finally, the trial that provided the largest number of patients found that a portion of patients randomized to TSV use did not use the vest according to the protocol, refused to wear the vest or did not wear the vest for other reasons and this introduces potential bias.

## CONCLUSION

This meta-analysis suggests that the use of a TSV after cardiac surgery could potentially be associated with a reduction in sternal wound complications. However, despite the significant treatment effect in the available studies, the evidence is not solid enough to provide strong practice recommendations.

## Supplementary Material

ivae055_Supplementary_Data

## Data Availability

The data underlying this article are available in the article and in its online supplementary material.

## ABBREVIATIONS

CABG: Coronary artery bypass graft
CDC: Centers for Disease Control and Prevention
CI: Confidence interval
COPD: Chronic obstructive pulmonary disease
DSWI: Deep sternal wound infection
LOS: Length of stay
OR: Odds ratio
PVD: Peripheral vascular disease
RCTs: Randomized clinical trials
SMD: Standardized mean difference
SWI: Sternal wound infection
TSA: Trial sequential analysis
TSV: Thorax support vest
