# Supplementary material for: Thorax support vest to prevent sternal wound infections in cardiac surgery patients—a systematic review and meta-analysis
Source: Interdiscip Cardiovasc Thorac Surg. 2024 Mar 26;38(4):ivae055. doi: 10.1093/icvts/ivae055 (PMC11035004; doi:10.1093/icvts/ivae055)

**Supplementary Material**

**Supplementary Table 1.** Search strategy for Ovid MEDLINE.

**Supplementary Table 2.** Individual studies’ eligibility criteria.

**Supplementary Table 3.** Risk of bias assessment using the Cochrane risk-of-bias tool for randomized trials (RoB 2).

**Supplementary Table 4.** Demographics of included patients from the selected studies.

**Supplementary Table 5.** Meta-regression for the primary endpoint (deep sternal wound infection).

**Supplementary Figure 1.** Leave-one-out analysis for the primary endpoint (deep wound sternal infection).

**Supplementary Figure 2.** Funnel for the primary endpoint (deep wound sternal infection).

**Supplementary Table 1.** Search strategy for Ovid MEDLINE (part 1).

| **Line #** | **Search** | **# of results** |
| --- | --- | --- |
| 1 | Braces/ or Orthotic Devices/ or External Fixators/ or *Equipment Design/ | 24614 |
| 2 | ((external support or chest support or stern* support or external stern* or thorax or thorax support or sternal stabilization or thorax stabilization or external supportive stern*) adj1 (device* or corset* or vest* or bandage*)).tw. | 34 |
| 3 | (Stern-E-Fix or SanThorax or Posthorax or stern* external fixation or elastic thorax bandage).tw. | 9 |
| 4 | or/1-3 | 24648 |
| 5 | Sternotomy/ or Sternum/su [Surgery] | 7300 |
| 6 | (sternotomy or sternotomies).tw. | 11377 |
| 7 | Cardiac Surgical Procedures/ or (cardiac surg*or heart surg* or heart valve surgery or cardiac operation* or heart operation* or cardiosurgery or myocardial resection or cardiothoracic surg* or cardiothoracic operation*).tw. | 67445 |
| 8 | Mitral Valve Annuloplasty/ | 1935 |
| 9 | ((bicuspid cardiac valve or bicuspid cardiac valvular or bicuspid heart valve or bicuspid heart valvular or bicuspid or bicuspid valve or bicuspid valvular or left atrioventricular cardiac valve or left atrioventricular heart valve or left atrioventricular valvular or mitral cardiac valve or mitral cardiac valvular or mitral heart valve or mitral heart valvular or mitral or mitral valvular) adj2 (annuloplast* or repair or replacement)).tw. | 15409 |
| 10 | Coronary Artery Bypass/ or Coronary Artery Bypass, Off-Pump/ | 55350 |
| 11 | (coronary adj2 (bypass* or graft* or surger*)).tw. | 57339 |
| 12 | (CABG or aorticocoronary anastomosis or total arterial revasculari*ation* or multiple arterial revasculari*ation*).tw. | 20914 |
| 13 | Heart Transplantation/ or (heart transplantation* or heart transplant or heart grafting* or cardiac transplantation* or cardiac transplant).tw. | 48509 |
| 14 | Cardiomyoplasty/ or (cardiomyoplasty or cardiomyoplasties).tw. | 997 |
| 15 | Heart Valve Prosthesis Implantation/ or (heart valve prosthesis implantation or heart valve prosthesis implant).tw. | 27460 |
| 16 | Myocardial Revascularization/ or (cardiac muscle revascularisation or cardiac muscle revascularization or coronary revascularisation or coronary revascularization or heart muscle revascularisation or heart myocardium revascularisation or heart revascularisation or heart revascularization or internal mammary arterial anastomosis or internal mammary arterial implantation or internal mammary artery anastomosis or internal mammary artery graft or internal mammary artery implant or internal mammary artery implantation or internal mammary-coronary artery anastomosis or Coronary Internal Mammary Artery Anastomosis or myocardial revascularisation or myocardial revascularization or myocardium revascularisation or myocardium revascularization or transmyocardial laser revascularisation or transmyocardial laser revascularization or vineberg operation).tw. | 22451 |

**Supplementary Table 1.** Search strategy for Ovid MEDLINE (part 2).

| **Line #** | **Search** | **# of results** |
| --- | --- | --- |
| 17 | Cardiac Valve Annuloplasty/ or (Cardiac Valve Annuloplasty or Cardiac Valve Annuloplasties or Valvular Annuloplasties or Valvular Annuloplasty or Heart Valve Annuloplasty or Heart Valve Annuloplasties or Cardiac Valve Annulus Repair or Heart Valve Annulus Repair or Cardiac Valve Annular Repair or Heart Valve Annular Repair or Cardiac Valve Annular Reduction or Cardiac Valve Annulus Shortening or Cardiac Valve Annulus Reduction).tw. | 1001 |
| 18 | (Aortic Valve Repair or Aortic Valve Replacement or aorta valve replacement or aorta valve transplantation or aortic valve transplantation or aortic valve xenotransplantation).tw. | 22773 |
| 19 | (tricuspid valve repair or tricuspid valve replacement or tricuspid valve transplantation).tw. | 2090 |
| 20 | Heart-Assist Devices/ or (heart assist device* or heart assist pump* or vascular assist device* or artificial ventricle* or ventricle assist device* or artificial heart ventricle*).tw. | 18080 |
| 21 | Transmyocardial Laser Revascularization/ or (transmyocardial laser revascularization or trans-myocardial laser revascularization or transmyocardial laser revascularisation or trans-myocardial laser revascularisation).tw. | 500 |
| 22 | or/5-21 | 264420 |
| 23 | 4 and 22 | 184 |

**Supplementary Table 2.** Individual studies’ eligibility criteria.

| Study | Eligibility Criteria |
| --- | --- |
| Caimmi | Patients at high risk for wound infections |
| Celik | Patients who underwent general cardiac surgery and sub-analysis with patients with moderate/severe COPD |
| Gorlitzer | Patients at high risk for wound infections |
| Tewarie | Patients at high risk for wound infections |

COPD= chornic obstructive pulmonary disease.

**Supplementary Table 3.** Risk of bias assessment using the Cochrane risk-of-bias tool for randomized trials (RoB 2).

| **Author** | **Year of publication** | **RANDOMIZATION PROCESS** | **ASSIGNEMENT TO INTERVENTION** | **ADHERING TO INTERVENTION** | **BLIDING OF INTERVENTION** | **MISSING OUTCOME** | **MEASUREMENT OF OUTCOME** | **SELECTION OF REPORTING RESULTS** | **OTHER SOURCES**  **OF BIAS** |
| --- | --- | --- | --- | --- | --- | --- | --- | --- | --- |
| Caimmi | 2016 | + | + | + | - | + | + | + | + |
| Celik | 2011 | + | + | + | - | +/- | + | + | + |
| Gorlitzer | 2013 | + | + | + | - | + | + | + | + |
| Tewarie | 2012 | +/- | + | + | - | + | + | + | + |
|  | | | | + | Low Risk | | | | |
|  |  |  |  | +/- | Some Concern | | | | |
|  |  |  |  | - | High Risk | | | | |

**Supplementary Table 4.** Demographics of included patients from the selected studies

| Study | Age  (mean±SD) | | Female (%) | | BMI  (mean±SD) | | | DM (%) | | Smoking (%) | | | Chronic renal failure (%) | | COPD (%) | | PVD (%) | | Prior MI (%) | |
| --- | --- | --- | --- | --- | --- | --- | --- | --- | --- | --- | --- | --- | --- | --- | --- | --- | --- | --- | --- | --- |
|  | **Vest** | **No**  **Vest** | **Vest** | **No Vest** | **Vest** | **No Vest** | **Vest** | | **No**  **Vest** | | **Vest** | **No**  **Vest** | **Vest** | **No Vest** | **Vest** | **No Vest** | **Vest** | **No**  **Vest** | **Vest** | **No**  **Vest** |
| Caimmi | 67.5±12.0 | 70.4±14.0 | 30.0 | 35.0 | 30.0±11.1 | 28.0±8.2 | NR | | NR | | NR | NR | NR | NR | 37 | 39 | NR | NR | NR | NR |
| Celik | 66.34±7.1 | 68.44±9.2 | 34.0 | 32.0 | NR | NR | 44.0 | | 47.0 | | NR | NR | 8.0 | 10.0 | 63.0 | 50.4 | 27.0 | 23.0 | NR | NR |
| Gorlitzer | 67.5±10.5 | 67.5±10.9 | 70.8 | 69.2 | 27.9±5.8 | 29.9±17.1 | 32.0 | | 29.5 | | NR | NR | 10.8 | 10.3 | 19.7 | 19.7 | 11.8 | 11.1 | 25.7 | 22.6 |
| Tewarie | 63.9±10.9 | 65.9±10.6 | NR | NR | NR | NR | 48.5 | | 45.8 | | NR | NR | 9.5 | 14.9 | 60.5 | 59.6 | 40.0 | 38.3 | NR | NR |

BMI= body-mass-index, CABG= coronary artery bypass grafting; CVA= cerebrovascular accident; DM= diabetes; HP= hypertension; MI= myocardial infarction; NR= not reported; SD= standard deviation; PVD= peripheral vascular disease.

**Supplementary Table 5.** Meta-regression for the primary endpoint (deep sternal wound infection).

| Variable | Beta ± SE, p- value |
| --- | --- |
| Age, mean | 0.2541 ± 0.4283, p= 0.65 |
| Chronic renal failure, % | 0.0599 ± 0.6238, p= 0.93 |
| COPD, % | -0.0272 ± 0.0090, p= 0.20 |
| PVD, % | -0.0378 ± 0.020, p= 0.31 |

COPD= chronic obstructive pulmonary disease, PVD= peripheral vascular disease.

**Supplementary Figure 1.** Leave-one-out analysis for the primary endpoint (deep wound sternal infection).


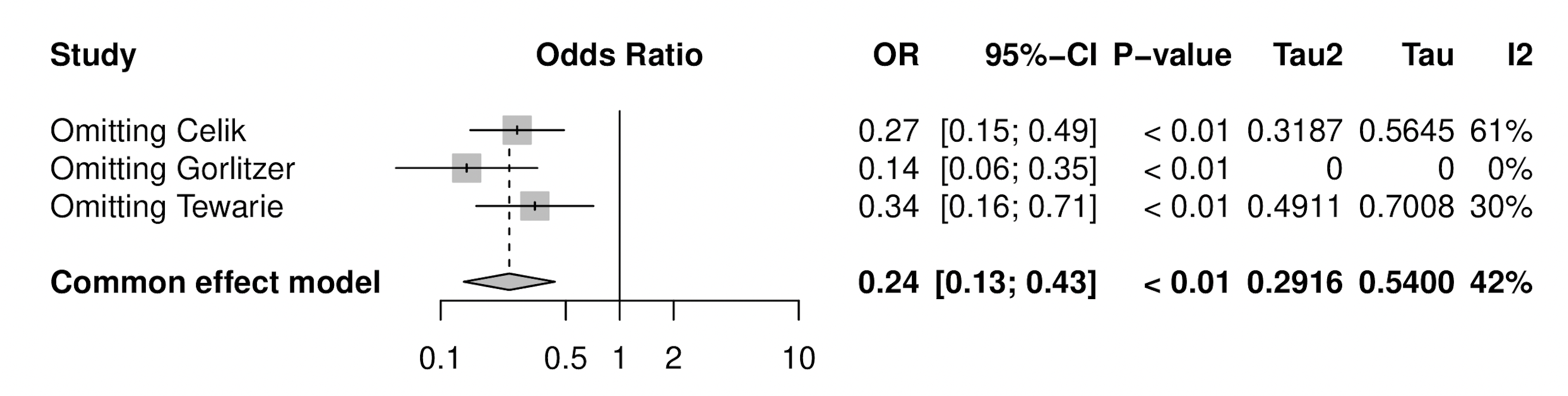


**Supplementary Figure 2.** Funnel for the primary endpoint (deep wound sternal infection).


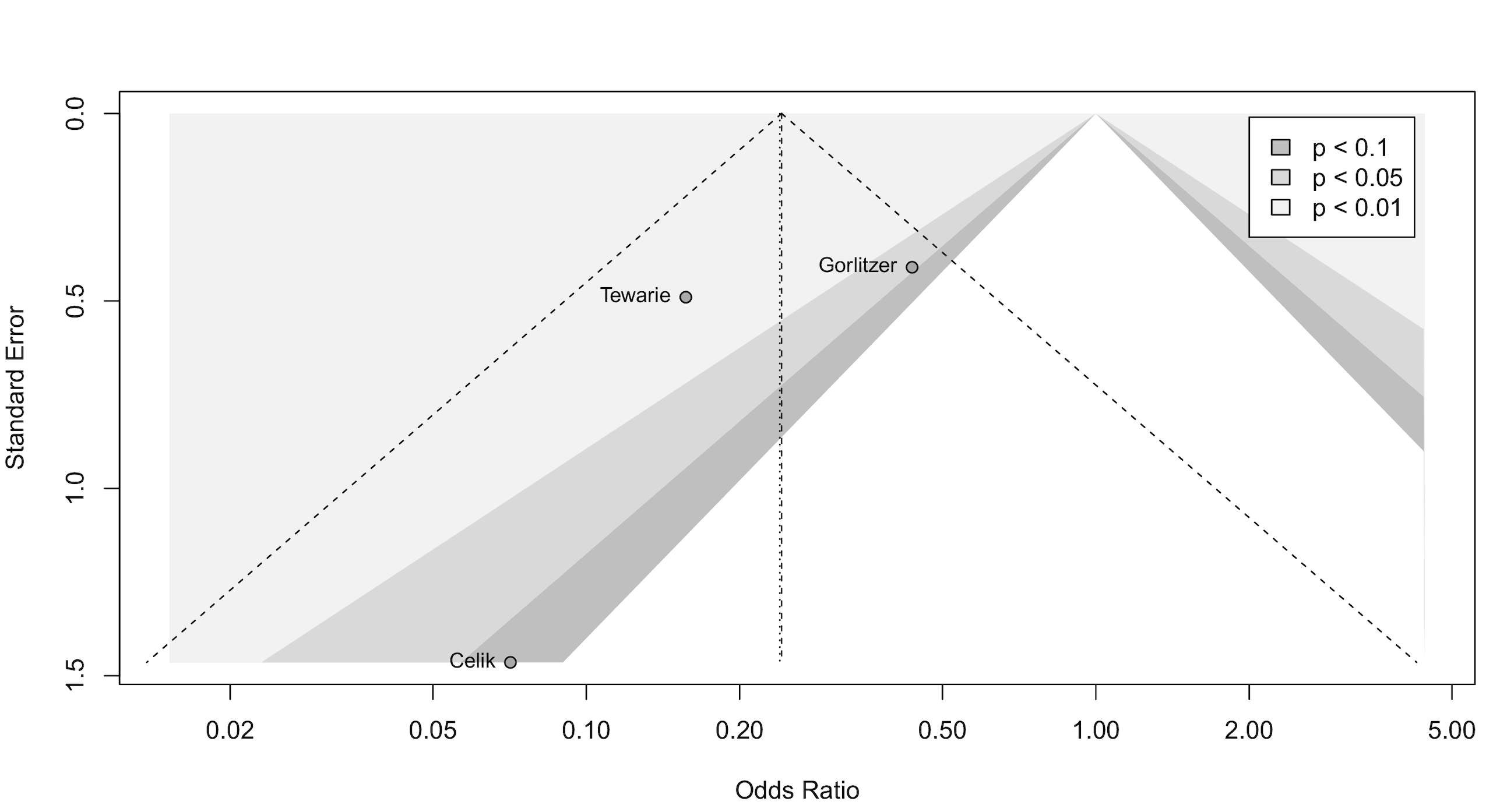

Supplement: ivae055_Supplementary_Data [file ivae055_supplementary_data.docx]
